# Water and Latrine Services and Associated Factors among Residents of Negele Town, Southeast Ethiopia: A Cross-Sectional Study

**DOI:** 10.1155/2022/1203514

**Published:** 2022-01-25

**Authors:** Diriba Temesgen Dagaga, Girma Deboch Geleta

**Affiliations:** ^1^Madda Walabu University, College of Natural and Computational Sciences, Department of Biology, P. O. Box 247, Bale Robe, Ethiopia; ^2^Negele College Preparatory High School, Negele Town, Ethiopia

## Abstract

**Background:**

Access to at least a basic water service, improved sanitation, and hygiene contribute to the human health and socioeconomic development of a country. This study was conducted to assess the water and latrine service coverage and related factors among dwellers of Negele town, southeast Ethiopia.

**Method:**

Two Kebeles (small administrative units) were randomly selected from each of the three zones of the town to collect data via questionnaires from randomly selected household heads (380), interviews of purposely selected key informants (40), and personal observations employing a cross-sectional survey design from March to May 2018. A Chi-square test was conducted to examine the association between various demographic factors and having latrine/tap water.

**Result:**

Latrine coverage of the town was low (45%) mainly due to shortage of land or funds and expansion of illegal houses. The available latrines were poor in hygiene. Water service (solely tap water) coverage was very low (7.6%) as a result of deficient water sources and nonfunctioning pipelines. The zones, age, educational status, marital status, and family size of the participants were statistically significantly associated with having latrine or tap water, *p* < 0.05. The administration of the town has planned to build four public toilets and raise its water supply coverage to about 70% by 2019/2020.

**Conclusion:**

Coverage of latrine and water services of Negele town were so low, implying that it is not on track to achieve the United Nations (UN) sustainable development goal target 6.1 and 6.2. The administration of the town should provide land to residents and search for fund sources for the construction of standardized private and public toilets. Utilizing various water sources, maintaining nonfunctional, and constructing new pipelines should be promoted to improve the water service coverage of the town targeting the national and UN sustainable development goals.

## 1. Background

Access to potable water and sanitation facilities like latrines is a basic human right and is related to social and psychological well-being, public health, socioeconomic development, and environmental sustainability [1]. However, millions of people living in developing countries lack access to such facilities due to fast population growth, poor service provision, and poor economic and educational status. They are conditioned to practice open defecation, expend much time and energy in fetching water, and tend to suffer from and die of a wide range of preventable diseases. Recognizing these, the United Nations [2] set the sustainable development goal target 6.1 (universal access to safe water) and 6.2 (universal access to sanitation) by 2030.

Although the country should work to achieve the United Nations development goals, open defecation has been commonly practiced throughout Ethiopia, for instance, by 28 million people in 2015, due to lack of hygiene awareness, adequate policy, and income [3]. Although the information is lacking at regional level in many cases and the national magnitude varies in different reports, the national estimated coverage of basic drinking water and basic sanitation in Ethiopia was 41% and 7%, respectively, in 2017 [4]. This showed that the country had not achieved the targeted 100% improved hygiene and sanitation coverage by 2015 and the country was not on the correct track to successfully extend safe water supply to 98% rural and 100% urban dwellers by 2020 via Water, sanitation, and hygiene (WASH) program [5]. Open defecation and lack of access to safe water have likely contributed to the wide prevalence of common waterborne diseases, and some recently emerged life-threatening Acute Watery Diarrhea (48,814 cases and 880 deaths in 2017) and cholera (6,578 cases and 56 deaths in 2017) as described by WHO [6] and Tesfay and Biru [7]), respectively, across the country.

As a developing country, Ethiopia has experienced rapid urban expansion while water and sanitary services are insufficient in many cases. Water and sanitary service coverage of an area need to be determined via scientific inquiry as they have environmental and population health risk implications and serve as an input to take appropriate measures. Such studies have been conducted in some parts of Ethiopia. Awoke and Muche [8] reported a 58.4% latrine coverage in Bahir Dar Zuria District, northwestern Ethiopia. Admassie and Debebe [9] indicated that about 68% and 95% of inhabitants of Wolaita Sodo Town (southern Ethiopia) had access to improved water supply and latrines, respectively. According to WASH [10], 31.2% of participants of Gonji Kolela Woreda in West Gojjam Zone, northwestern Ethiopia, were using either river or unprotected springs for their home consumption. Tesema [11] reported possession of latrines by 89% of households in Diretiyara, eastern Ethiopia. Similarly, Dagnew et al. [12] reported latrine coverage of 27.5% for Chiro Zuria District, eastern Ethiopia. These studies covered limited areas of the country with greatly varied results, implying the need to expand the study to other regions to get dependable information on water and sanitary service coverage of the regions and that of the country as a whole. Research based information can assist to develop appropriate policies to improve water and sanitary service coverage.

This study was conducted at Negele town, southeast Ethiopia. Our preliminary survey indicated that a portion of its residents have access to latrines and water service, whereas others practice open defecation and use unsafe water sources. However, a good estimation of the number residents of the town that have access to latrine and water service was absent due to the lack of previous studies at the town. This can affect the effectiveness of plan and policy that the administration of the town may develop to improve the coverage of water and sanitation services. Thus, the study was initiated to provide baseline data on latrine and water service coverage and associated factors of the town for improvement by relevant authorities to achieve the national and UN (United Nations) goals concerning access to safe water and sanitation.

## 2. Methods

### 2.1. Description of the Study Area

This research was conducted at Negele town, located in Guna district, Arsi zone, Oromia region, southeast Ethiopia (7°21′N; 38°42′E). The town was established on a landscape of 300 hectares in 1984. It is located 204 km away from Addis Ababa, the capital city of Ethiopia. Negele town experiences a mild climate with mean annual temperature and rainfall ranging from 12 to 23°C and 700–1300 mm, respectively. The town has been administered at the municipality level since 2000 and has its administrative structure led by a mayor. The administration of the town estimated its population to be 22,578 in 2017. The town is demarcated by different rural “Kebeles” (the lowest administrative unit in Ethiopia): Amuma-Arago in the east, Nano Jawi in the west, Nano Hecho in the north, and Cire Anole in the south. It has a primary school, a secondary school, and a health center. The economic activity of its surrounding population is predominantly agriculture, comprising farming and cattle breeding.

### 2.2. Study Design and Population Sampling

A community-based cross-sectional qualitative and quantitative descriptive survey was conducted at Negele town from March 24 to May 31, 2018. The town is divided into three sub-administrative zones: Central zone (Central Hindy, Center of town, and Central villages), Eastern zone (Najate, Sheep site, and East Hindy villages), and Western zone (Western Hindy, Mosque area, and Secondary school area villages). During the study, there were 4095 households; 2631 and 1464 represented by males and females, respectively, as family heads that lead a family, take major social responsibilities, and make decisions about the family. The study targeted 2729 (1598 males and 1131 females) households living within six randomly selected villages (Table 1), two villages from each zone, as they were relatively homogenous according to the preliminary information obtained from the town's administrative office. Households were randomly sampled and the sample size (369) was determined according to Naing et al. [14]. To minimize errors arising from attrition, 10% of the sample size was added making the total sample to be 406 (244 males + 162 females). Then, the proportional sample size method was applied to allocate the number of participant households to each village. Both sexes were encouraged to participate in the study. As the overall coverage of latrine and water service (*P*) was unknown for the study area, the maximum national coverage of environmental sanitation with the main component of latrine coverage for Ethiopia (60%) was considered at a 95% confidence interval (*Z*) and a 5% degree of accuracy (*d*). Individuals below 18 or above 80 years were excluded to gather data from matured active individuals.

### 2.3. Data Gathering Tools and Procedures

A recognizance survey was conducted before the actual study from 24 to 30 March 2018 to sketch out the overall status of latrine and water service coverage. WHO and UNICEF [15] were referred to in the preparation of certain portions of data-gathering tools (observation, interview, and questionnaire) (See https://downloads.hindawi.com/journals/JEPH/2022/1203514.f1.docx file for details), but most of them were prepared by the authors based on the prevailing community practices, the resources, and information accessible to the residents.

Latrines were observed for the presence of doors, roofs, hole covers, and water supply, while households were filling questionnaires according to the prepared observational checklist. Observations were made after obtaining the consent of the participants. Water fetching processes and various fields for open defecation sites were also observed.

A structured questionnaire was administered to gather data related to the households' sociodemographic characteristics, occupation, educational level, source of water, presence or absence of latrines, latrine doors, hole cover, and roof, where they defecate, and what problems they have faced in case they lacked a latrine, the presence of anybody who advised them to construct a latrine, whether they have a plan to construct a latrine shortly, the number of people using a latrine, the distance of the latrine from the kitchen, availability of water to clean the latrine and sewerage service to clean the latrine, measure (*s*) taken when the latrine became full, source of water, treatment(s) undertaken for nontap water before drinking, and exposure to waterborne diseases. Interview was held with 40 purposively selected key informants (head of administration of the town, Kebele officials, water and health sector workers) regarding their sociodemographic attributes, presence of public latrines, factors affecting latrine and water service coverage, consequences of latrine shortage, presence of a plan and its target to improve the latrine and water service coverage of the town. As a key informant, the chief administrator of the town was also requested for any relevant additional information that he would like to add. Questions were prepared in English and translated into a local language (Afaan Oromo). Necessary orientation was provided to facilitate the process of filling out the questionnaire or responding to the interview questions. Questions were presented in the same wording and the same order to all participants.

### 2.4. Data Validity and Analysis

Before dispatching, questionnaires were tested on 25 purposely selected potential participants (19 males and 6 females) and the Cronbach's alpha score was found to be 0.799 and considered reliable according to Gliem and Gliem [16]. Collected data were checked for completeness, readability, or error. Descriptive and inferential analyses of quantitative data were performed employing Statistical Package for Social Science (SPSS; version 20) and results were presented in the form of frequency and percentage tables. A Chi-square test was conducted to see the association between various determinant factors and having latrine or tap water at *p* ≤ 0.05 for statistical significance.

Narrative analysis was applied for qualitative data obtained via interviews, observation, and open-ended questionnaires. Data were repeatedly read and well understood, sorted by question/topic, and organized into coherent categories followed by interpretation.

## 3. Results

### 3.1. Demographic Characteristics of the Participants

#### 3.1.1. Demographic Characteristics of Household Heads

Out of 406, 380 (93.6%) households' heads properly filled and returned the questionnaires. Most of them were young (18–30 years), Muslims (86%), and married (68%) (Table 2).

Regarding educational status (Table 2), 118 (31.05%) of the household heads had never attended formal education, whereas 186 (51.6%) and 44 (11.57%) of them had attended primary (grade 1–-8) and secondary level (grade 9–-12). Only 22 (5.78%) of the participant household heads completed secondary school (grade 12). The majority of the participant household heads (277; 59.7%) had up to 4 household members, whereas 108 (28.4%) and 45 (11.8%) of them had 5–-10 and over 10 family size, respectively. The participant household heads have engaged in different types of jobs. They were predominantly farmers and merchants.

#### 3.1.2. Demographic Characteristics of the Key Informants

Forty key informants with sociodemographic features indicated in Table 3 were properly interviewed. Most of the key informants were health workers, males, Muslims, married, diploma holders, and found within the age range of 18–40 years.

### 3.2. Latrine Coverage, Associated Facilities, and Usage

Only 45.3% of the household head participants indicated that they had latrines (Table 4). This was also confirmed by the authors' observation checklist data. Latrine coverage was lesser in the eastern zone of the town compared to central and western zones. Moreover, over 60% of the household head participants who had latrines indicated that a latrine was used by more than 5 people.

**Figure 1:**
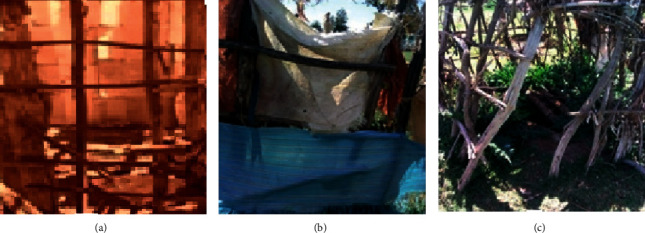
Appearances of some latrines of the participants of Negele town in 2018 (photo by Girma Deboch, 2018). (a) Incomplete wall with no roof, (b) surrounded by sacks to avoid exposure but with no roof, and (c) simple fence surrounded with no roof.

The majority of the available toilets were built without skill and technology and were found wanting in hygienic features like water supply, hole cover, roofs, doors (Figure 1), and the recommended distance from kitchen. Fifty-seven (33.1%), 54 (31.4%), 18 (10.5%), and 43 (25%) of household heads owning latrines said that their latrines are 3–5 m, 2–3 m, 6 m, and greater than 6 m away from kitchens, respectively. Data collected via an observation checklist revealed that 35.5% of households' latrine was located at a distance less than 6 m from the kitchen (Table 5) compared to the value (45.5%) obtained via the questionnaire (Table 4).

More than half (52.3%) of the households' heads replied that their latrine lacked roofs. Roughly, 50% of households' heads from the central and western zones indicated the presence of latrine roofs, whereas only 38% of eastern zone households' heads indicated the presence of latrine roofs. Fifty-three percent and 100% of the households' heads indicated the absence of latrine doors and hole cover, respectively (Table 4). Data on latrine roof, wall, and hole cover obtained via observation (Table 5) matched that of the questionnaire. The roofs were made from metal sheet, or wood covered with grass, plastic, or fertilizer sacks. Similarly, the walls were made from fenced wood or plastic, or other material supported by wood (Figure 1) to prevent exposure of the users or entry of animals. Residents have usually been advised by health extension workers to put a sheet of metal or wood on the small hole of the latrine (called “hole cover” in this article) to protect flies, but none of them had done it.

The entire latrine-owned households' heads pointed out the lack of water to clean their latrines (also confirmed via observation) and sewerage services to clean their latrines when they became full. The lack of sewerage service to collect sewage when toilets become full had forced 70% and 30% of latrine owning household heads to dig new toilets and to drain their latrines into the environment, respectively (Table 4). Moreover, only 6 (3.48%) latrine owning households' heads perceived their latrines as clean and good for health in contrast to 166 (96.51%) that considered their latrines as unclean, usually dirty, and unhealthy. The authors' observations supported the latter as indicated in Figure 1.

### 3.3. Absence of Latrine and Associated Factors

**Figure 2:**
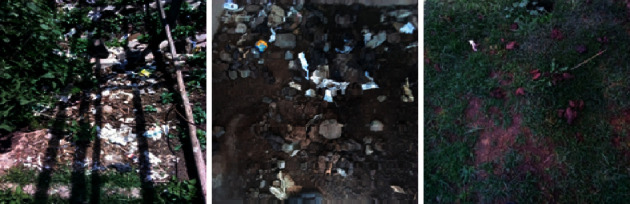
Some open defecation sites in Negele town (photo by Girma Deboch, 2018).

Shortage of income and land was the reason cited by the majority of households' heads for not having latrines (Table 6). However, most of the interviewed key informants pointed out the low involvement of the administration of the town, low residents' awareness-/attitude-related issues as the main factor that had hindered the residents from building toilets (Table 7). Households latrine lacking were used to defecate in open fields (56; 26.92%), in a bush (54; 26%), in house compounds (80; 38.5%), or in any place as needed (18; 8.65%) (Table 6; Figure 2), as there were no public toilets as an alternate. Similarly, 100% and 43% of the key informants confirmed the absence of a public toilet and the existence of open defecation practices in the town, respectively (Table 7). The authors observed no public toilets in the town during the study. The practice of open defecation in different parts of the town was also noticed by the investigators during the field survey as a result of the shortage of private latrines together with the lack of public latrines (Figure 2). However, it was not possible to enumerate the people that practiced open defecation. The chief administrator of the town stressed that most of the houses that lacked latrines were built by people living in the surrounding rural areas and that the authorities found it difficult to create an awareness to avoid open defecation. About 50% of the households' heads replied that they had been advised to construct toilets either by health extension workers or by local leaders (Table 6).

Household heads lacking latrines expressed that they had been suffering from lack of safety, illness, pollution of living area, and moving out in the dark for defecation. Similarly, 35% and 23% of the key informants expressed the prevalence of waterborne health problems and environmental pollution in the town, respectively, due to lower latrine coverage (Table 7). However, 67% of household heads lacking toilets expressed that they had no plans to construct latrines in the near future due to income, land, or information constraints as expressed earlier. The authors also noticed that most of the houses in the town were built on small plots of land without following the plan of the town and allocating space to construct latrines. The chief administrator of the town, a key informant, pointed out that the administration of the town had planned to build four public toilets by 2019/2020. However, two-third of the key informants had no information regarding the plan of the administration of the town at all (Table 7).

### 3.4. Water Service Coverage and Associated Conditions

**Figure 3:**
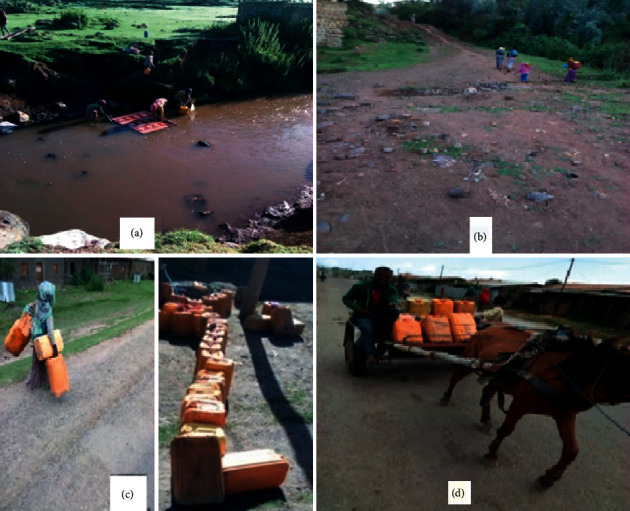
Fetching water from Nano River and transporting for longer distances on the back of people (a, b) and fetching water from the private tap with long waiting line (c) and transporting using a horse (d) (photo by Girma Deboch, 2018).

Only 29 household heads, all from the central zone of the town, replied that they had private tap water (Table 8). Few household heads said that they used to buy others' tap water expending much of their time lining up and their energy in carrying water for longer distances, whereas the majority of them replied that they were using unprotected water sources including ponds/rain and river water, particularly from Nano River traveling 3–5 km to the north of the town. These were also observed by the investigators (Figure 3), but it was not possible to count the number of people using each water source, so the numbers relied on the response of the household heads (Table 8). The area of the Nano River is mountainous making it somewhat difficult to fetch water from it. Moreover, using the river water directly for drinking could have created health problems as 72.6% of the household head participants replied that they directly use nontap water without boiling or chemical treatment (Table 8). Forty-five percent of the household heads said that they had no knowledge about the effect of impure water on health, but 51% of them said they or their families had contracted waterborne diseases.

**Figure 4:**
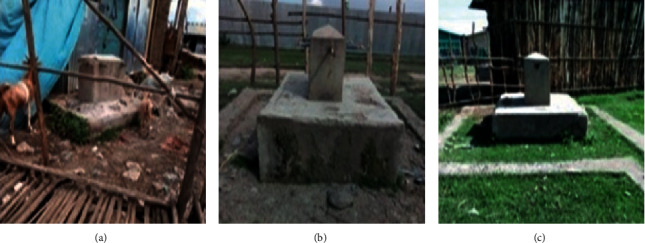
Nonfunctional public pipes (a–c) in the study area (photo by Girma Deboch, 2018).

Forty-five percent of the key informants expressed nonfunctioning public water pipes (Figure 4) as a factor for reduced water supply to the town. Public pipes were constructed in the town in 2010 at different places but became nonfunctional from 2013 to the time of the investigation.

The observational survey (Table 5) also revealed the presence of only a few private taps with infrequent and insufficient water. Moreover, all public pipes were not functioning during the investigation (Figure 4). Several key informants also mentioned low involvement of the administration, inadequate quantity of water from the source, and lack of storage tanks as contributing factors to the low water service coverage of the town. However, the chief administrator of the town, also a key informant, stressed the inadequacy of water as a major limiting factor for the provision of sufficient water to the town. The chief administrator also expressed that the town had planned to build water tankers and public water pipes across the town to raise its water supply coverage to about 70% by 2019/2020. Only one-third of the key informants knew the existence of the plan although they were not sure about the planned percentage of water service coverage improvement as they put various ranges (Table 7).

### 3.5. Factors Associated with Having Latrine and Tap Water

The zonal sites, age, educational status, marital status, and family size of the households' head participants were found to be statically significantly associated with having latrines, *p* < 0.05 (Table 9). The proportion of household head participants having latrines increased with the increase in age. More proportion of household head participants who had completed grade 12 possessed latrines compared to those with primary or secondary school education. However, a higher proportion of household head participants with no formal education owned latrines. Marital status was also found to be significantly associated with having a latrine (*X*^2^ = 8.891; *p* ≤ 0.05), where less proportion of married participants possessed latrines in contrast to single or widowed ones. Similarly, family size was significantly associated with having latrines (*X*^2^ = 14.23; *p* ≤ 0.01), whereas more portions of participants with larger family sizes tend to possess latrines.

The study indicated the lack of significant association between having a latrine and sex, religion, or type of occupation, *p* < 0.05 (Table 9).

Factors that showed significant association with having a latrine (zone, age, educational status, marital status, and family size) were also found to be significantly associated with having tap water (Table 10). Only participants from the central zone owned tap water. Similar to latrine ownership, the proportion of tap water ownership had increased with the increase in the age of participants. Educational status was also significantly associated with having tap water (*X*^2^ = 11.882, *p* ≤ 0.01). Similar to latrine ownership, tap water ownership showed no significant association with sex, religion, or type of occupation of the participants as their *p* values were ≥0.05 (Table 10).

## 4. Discussion

The overall latrine coverage of the town (45.3%) was lower than that reported for some towns in Ethiopia including Dukem town (70.1%; [17]) and Wolaita Sodo town (91%; [9]), for Ilu Aba Bor Zone (88.2%; [18]) and the overall Oromia region (72.7%; [19]) in which the town of study (Negele town) is found. The variations might be due to variations in awareness level and socio-economic status of the residents among others.

Besides lower coverage, the latrines were poor in hygiene. With a slight discrepancy in data collected via questionnaire-based participants' estimation and via measurement during observation, over one-third of the existing latrines were closer than the minimum recommended distance (6 m) from a kitchen according to WHO [5]. Thus, it is easy for the bad odor to reach living rooms and for flies to carry pathogens to the kitchen where food is prepared and kept. Similarly, in a different study, about 76% of participants from Nepal indicated that latrine distances from their homes were less than 6 m [20].

The absence of latrine roofs (52.3%), doors (53%), and hole covers (100%) could promote the invasion and breeding of flies leading to disease dissemination. Even the existing roofs were made from torn plastic or other materials which cannot protect from flies or rain. Doors did not fit well and extended only up to half the height of the latrine merely to hide the users in some cases, implying the possibility of free invasion of flies. The participants had not accepted or practiced the usual advice of health extension workers to put a sheet of metal or wood (called hole cover in this article) on the small hole of the latrine to protect flies and reduce bad odor. This showed the necessity for further awareness creation and follow-ups. However, Yimam et al. [21] reported the presence of latrine hole cover in 47.6% of the latrines in Dembia town, northwestern Ethiopia.

Lack of cleaning water not only causes bad odor to reach homes but also hinders handwashing activity after using the toilet. Lack of sewerage service to collect sewage from filled latrines had forced the majority of the household heads (70%) to dig new toilets which incur additional expenses. It had also forced some of the household heads (30%) to drain the filled toilet to the environment which could affect human health and environmental sanitation. In many towns in Ethiopia, residents are not allowed to connect their latrines to wastewater channels as treatment is not practiced; rather, vehicles are available to draw out latrine waste and dispose of the waste somewhere else. In areas where such a facility is unavailable, full latrines need to be abandoned leading to land or money constraints to build new ones.

Perception of their latrine as clean, standardized, and good for health by a few participants (3.5%) in the absence of good roofs, doors, and water for cleaning reflects their lack of good knowledge of sanitation, implying the need to raise public awareness and provide technical assistance in building latrines. In a previous study, Dagnew et al. [12] reported that 13% of the participants were using improved latrines in Chiro Zuria District, eastern Ethiopia.

Lack of enough land and money was raised by households as the key factor that hindered them from building their latrines, leading them to defecate in open fields, bush, and house compounds due to the lack of public toilet as an alternative. Similarly, households' capacity to finance the construction of home toilets was reported to be a fundamental factor in Wa Municipality of Ghana [22]. On the other hand, the key informants raised low involvement of the administration of the town, low residents' awareness-/attitude-related issues as the main factor that had hindered the residents from building their latrines. This shows that it is important to bring various sections of the society together to discuss and identify the key factors that hinder latrine construction to minimize or avoid open defecation, as it influences environmental sanitation, human health, and psychology [23]. The administration of the town should be committed to providing enough land, searching for funding sources, constructing public toilets, and raising public awareness to solve the problem. Special attention should be given to public awareness creation and mobilization as 67% and 50% of households lacking latrines expressed that they had no plan and did not get advice, respectively, to build a latrine. Moreover, Yimam et al. [21] indicated that 88.6% of the participants who had latrines were advised to construct latrines by health extension or community health agent personnel in a northwestern Ethiopian town, Dembia.

Some open defecation sites were observed in the town during the study, but it was not possible to count households practicing it as many of them went out at night or early in the morning. No area was specifically designed for open defecation, but sloppy or deep eroded areas that are unsuitable for constructing homes were used for that purpose.

The plan of the administration of Negele town to build four public toilets at different parts of the town by 2019/2020 may be considered as a starting step to improve sanitation of the town. However, it seems to be insufficient. Moreover, our current information (December 2021) from the residents revealed that the administration of the town had built a single public toilet near a newly established bus station. The administration should focus on the constraints raised by the participants to bring better improvement in sanitary coverage. The administration should also control illegal house construction whose dwellers were found to commonly practice open defections due to lack of latrines.

The water service coverage (7.64%) of the town was lower than other towns like Dukem (98.5%; [17]) and Wolaita Sodo (68%; [9]), and a rural district in western Ethiopia (70%; [24]) forcing most of the households to use unprotected Nano River water without heat or chemical treatment and thereby suffer from waterborne diseases. This implies that immediate action should be taken by the administration of the town and other concerned bodies to improve water service and create awareness of the community to boil or treat water using Wuha agar (chlorine-based water treatment solution) and Bishan gari (aluminum sulfate and calcium hypochlorite solution). These chemicals are available in the market and usually announced via mass media in different languages to be used to treat water at home to prevent waterborne diseases.

The administration of the town should work hard to effectively implement its plan of raising water service coverage to about 70% by 2019/2020. However, the plan should be communicated as several key informants had no information about the plan which might hold true for other residents. The plan should also include maintaining nonfunctional public water pipes, developing various water sources including groundwater and springs, and establishing/expanding water purification and storage facilities. The plan should also target achieving Ethiopia's plan of providing safe water to all urban dwellers by 2020 [23]. The commitment of the administration of the town should also be added as key informants raised its lower involvement contributing to lower water service coverage of the town. Currently (December 2021), the residents of the town confirmed that the administration of the town totally failed to raise the water service coverage of the town according to the plan as it has done nothing to build water tanks, public water pipes, or develop new water sources at the town.

Generally, the latrine and water service coverage of the town was found to be much lower than the National Millennium Development Goal (MDG) of Ethiopia that targeted 100% improved hygiene and sanitation by 2015 [25], although the country managed to improve sanitation coverage from just 8% (1990) to 71% (2015) and reduce open defecation from 44.3 million (1990) to 28.3 million (2015) [19]. This further indicates that the town/country is not on track to extend safe water supply to 98% and 100% of rural and city dwellers, respectively, by 2020 via WASH [5] and to achieve sustainable development goals targets 6.1—universal access to safe water and 6.2—universal access to sanitation by 2030 [2]. However, some recent reports indicated that various nations are on track towards achieving United Nation's sustainable development goals (UN's SDG). Pereira and Marques [26] reported the convergence of low- and middle-income countries towards achieving the UN's SDG 6, but at the expense of worsening the level of water crisis. Similarly, Pereira et al. [27] reported the convergence of the World Health Organization Member States regarding the United Nations' Sustainable Development Goal “Good health and well-being.”

The zonal sites, age, educational status, marital status, and family size of the participants were statically significantly associated with having a latrine (*p* < 0.05). Although other sources of information like religious education, mass media, and health extension workers exist, the possession of latrines by a higher proportion of g household participants who completed grade 12 compared to primary or secondary level revealed the influence of education on sanitation awareness of the participants to construct their latrine. Educational status was also significantly associated with the use of improved sanitation in Ethiopia [3,9]. Similarly, individuals who had completed high school demonstrated better latrine utilization than those without formal education [11]. However, a higher proportion of participants of the current report with no formal education also possessed a latrine, which could be related to increased age as most individuals with no formal education are usually older and the study showed more latrine ownership among aged individuals. This could be due to having large house compound areas and building latrines through time by elder residents as found in many towns in the country. Recently, urban administrations gave residents small areas of land (as small as 75 square meters) to build homes due to a rapid increase in population size following rural to urban migration, and the land surrounding the towns are farmlands owned by farmers. However, it is common to buy such farmlands illegally and build homes there. In some cases, towns and urban areas lack clear boundaries. Building illegal houses without latrines or legal houses with a latrine at different parts of the town could have led to variation in latrine coverage across the zones.

Statistically significant association of family size with having a latrine could be due to the fact that larger families could have higher potential to construct their latrine as certain members could be employed generating income. Regarding marital status, married individuals are expected to be independent of their family and build their own houses and latrines unless constrained by the shortage of funds or land described in the responses to the questionnaire.

In previous studies, “perception of building a toilet is expensive” was found to be associated with reduced toilet ownership among rural households in some eastern districts of Indonesia [28]. Education and household size were among the determinants of open defecation, which is related to the lack of latrine ownership in the Wa Municipality of Ghana [22]. Ajemu et al. [29] reported that promotion of Health Extension Workers, possession of private houses, and occupational status were more likely to be associated with latrine construction among some rural villages of Tigray region, northern Ethiopia.

Zones, age, educational status, marital status, and family size of the participants were statistically significantly associated with having tap water sources similar to latrine ownership. In Ethiopian towns, the central parts are usually the first to be established and get services like water and electricity. Old people that established the towns usually live in the central parts of towns where water services are better compared to other parts in most cases. Educational status can be related to income and information to get access to water service (tap water) as the data revealed that participants with no formal education were least in the proportion of owning tap water. The association between marital status or family size with having tap water could be due to the amount of daily water demand. Marriage increases the family size and larger families require more water and could have made a better effort to own tap water. Similarly, Gebremichael et al. [30] reported a significant association of households' drinking water sources with age, educational status, and family size in northwest Ethiopia. Earlier, Fortune and Sikod [31] indicated a significant association of distance of water sources and household size with the choice of drinking water source in Cameroon.

## 5. Conclusion

Latrine coverage of Negele town was low (≈45%) due to shortage of land and funds, low involvement of administration and residents, and the expansion of illegal houses. The existing latrines lacked hygiene features. Low coverage of private toilets together with the absence of public toilets in the town has led to widespread open defecation practices with potential and practical negative impacts on the health of the community and sanitation of the environment. The water service coverage (tap water) of the town was also very low (below 10%) due to inadequate water sources and the nonfunctionality of the existing water taps. The problems associated with lower latrine and water service coverage of the town seem to continue in the near future as most toilet-lacking households had no plans to construct it and the administration of the town failed to implement its plan; constructed one of the four planned public toilets and did nothing in the case of water service as of December 2021. The zonal sites, age, educational status, marital status, and family size of the participants were statistically significantly associated with having latrine or tap water; *p* < 0.05.

The administration of the town should provide land for latrine construction, construct a sufficient number of public toilets, should supply loans or search for aids as well as provide technical assistance for the construction of standardized private and public toilets. Searching for additional water sources like groundwater and maintaining nonfunctional and/or constructing new water pipes should be a point of focus of the administration of the town to improve the water service coverage of the town. Plans to improve latrine and water service coverage of the town should involve the residents and committed governmental/nongovernmental bodies taking the national and UN Sustainable Development Goals into account.

## Figures and Tables

**Table 1:** Study villages with their corresponding zones, target, and study households of Negele town during the study period (source: [13]).

No.	Zones	Total households	Selected villages	Target households	Sampled households (*n* = 406)			
					Frequency		Total	%
					Female	Male		
1	Central	1718	Center of town	590	34	54	88	21.67
			Central villages	555	36	46	82	20.20
2	Eastern	1353	Sheep site	382	23	34	57	14.04
			Najat	520	30	47	77	18.97
3	Western	1024	Western Hindy	417	24	38	62	15.27
			Mosque area	265	15	25	40	9.85
Total	3	4095	6	2729	162	244	406	100%

**Table 2:** Demographic characteristics of heads of households that participated in the study (*n* = 380).

Variable	Frequency (%)		
*Administration zones*	Female	Male	Overall
Central	68 (17.9)	98 (25.8)	166 (43.7)
Eastern	45 (11.8)	81 (21.3)	126 (33.2)
Western	25 (6.6)	63 (16.6)	88 (23.1)

*Age (years)*			
18–30	113 (29.7)	75 (19.7)	188 (49.5)
31–40	55 (14.5)	86 (22.6)	141 (37.1)
41–80	28 (7.4)	23 (6.1)	51 (13.4)

*Religion*			
Christian	28 (7.4)	40 (10.5)	68 (17.9)
Muslim	110 (28.9)	202 (53.2)	312 (82.1)

*Educational status*			
No formal education	41 (10.8)	77 (20.3)	118 (31.0)
primary (1–8)	66 (17.4)	130 (34.2)	196 (51.6)
Secondary (9–12)	19 (5)	25 (6.6)	44 (11.6)
>Grade 12	12 (3.2)	10 (2.6)	22 (5.8)

*Marital status*			
Single	27 (7.1)	73 (19.2)	100 (26.3)
Married	111 (29.2)	149 (39.2)	260 (68.4)
Widow	0 (0)	20 (5.3)	20 (5.3)
Divorced	0 (0)	0 (0)	0 (0)

*Family size*			
1–4	90 (23.7)	137 (36.1)	227 (59.7)
5–10	26 (6.8)	82 (21.6)	108 (28.5)
>10	22 (5.8)	23 (6.1)	45 (11.8)

*Occupation*			
Farmer	59 (15.5)	117 (30.8)	176 (46.3)
Merchant	29 (7.6)	91 (23.9)	120 (31.6)
Government employee	28 (7.4)	13 (3.4)	41 (10.8)
Daily laborer	14 (3.7)	0	14 (3.7)
Other	8 (2.1)	21 (5.5)	29 (7.6)

**Table 3:** Demographic features of the key informants (*n* = 40).

Variable	Occupation			
	Head of town administration	“Kebele” official	Water sector workers	Health workers
*Sex*				
Male	8	5	3	18
Female	1	0	0	5

*Age (years)*				
18–40	7	3	3	21
41–60	2	2	0	2
61–80	0	0	0	0

*Religion*				
Muslim	7	5	3	14
Christian	2	0	0	9
Other	0	0	0	0

*Marital status*				
Single	3	1	0	8
Married	6	4	3	15
Widow	0	0	0	0
Divorce	0	0	0	0

*Educational status*				
Grade 9–10	3	4	0	0
Grade 11–12	1	0	0	0
Certificate	0	0	0	0
Diploma	2	1	3	15
Degree	3	0	0	8
Other	0	0	0	0

**Table 4:** Responses of households of Negele town (*n* = 380) on latrine coverage and associated facilities.

Variable	Frequency (%) of participants per each zone			Overall (%)
	Center	East	West	
*Presence of own latrine* ^*∗*^				
Yes	82 (49.4)	40 (31.7)	50 (56.8)	172 (45.3)
No	84 (50.6)	86 (68.3)	38 (43.2)	208 (54.7)

*Number of users of a latrine*				
1–5	33 (40.2)	12 (30.0)	20 (40.0)	65 (37.8)
6–10	30 (36.6)	8 (20.0)	20 (40.0)	58 (33.7)
Greater than 10	19 (23.2)	20 (50)	10 (20.0)	49 (28.5)

*Distance of latrine from kitchen (m)*				
2–3	27 (32.9)	11 (27.5)	16 (32.0)	54 (31.4)
3–5	28 (34.1)	17 (42.5)	12 (24.0)	57 (33.1)
6	9 (11.0)	5 (12.5)	4 (8.0)	18 (10.5)
Greater than 6	18 (22.0)	7 (17.5)	18 (36.0)	43 (25.0)

*Presence of latrine roof*				
Yes	41 (50)	15 (37.5)	26 (52.0)	82 (47.7)
No	41 (50)	25 (62.5)	24 (48.0)	90 (52.3)

*Presence of latrine door*				
Yes	39 (47.6)	24 (60.0)	18 (36.0)	81 (47.1)
No	43 (52.4)	16 (40.0)	32 (64.0)	91 (52.9)

*Presence of latrine hole cover*				
Yes	0 (0)	0 (0)	0 (0)	0 (0)
No	82 (100)	40 (100)	50 (100)	172 (100)

*Presence of water for cleaning latrine*				
Yes	0 (0)	0 (0)	0 (0)	0 (0)
No	82 (100)	40 (1000	50 (100)	172 (100)

*Presence of sewerage to clean latrine*				
Yes	0 (0)	0 (0)	0 (0)	0 (0)
No	82 (100)	40 (100)	50 (100)	172 (100)

*Whether latrine became full and overflown earlier or not*				
Yes	36 (43.9)	9 (22.5)	12 (24.0)	57 (33.1)
No	46 (56.1)	31 (77.5)	38 (76.0)	115 (69.9)

*Measures taken when latrine was full*				
Digging new toilet	65 (79.3)	20 (50.0)	36 (72.0)	121 (70.3)
Drainage to environment	17 (20.7)	20 (50.0)	14 (28.0)	51 (29.7)

*Perception of one's latrine*				
Clean and good for health	5 (6.1)	0 (0)	1 2 (0)	6 (3.5)
Dirty and unsuitable for health	77 (93.9)	40 (100)	49 (98.0)	166 (96.5)

^*∗*^The responses to the first question were given by all household heads who participated in the study, whereas the responses to other questions in the table were provided by only households that possessed private latrines

**Table 5:** Observational checklist used to collect data from households (*n* = 380).

No.	What was observed (present/absent if applicable)	Yes (%)	No (%)	Remark
1	Household latrine	172 (45.3)	208 (54.7)	
A	Latrine door	81(47.1)	91 (52.9)	For households that had latrines
B	Latrine roof	82 (47.7)	90 (42.3)	
C	Latrine hole cover	0	172{100)	
D	Latrine water supply to clean	0	172(100)	
E	Distance of latrine from kitchen			
	<6 m	61 (35.5)	111 (64.5)	

2	Using public latrine	0	172 (100)	For households without latrine

3	Practicing open defecation ^*∗*^			Various parts of the town were observed

4	Drinking-water source (*n* = 380)			
A	Own private tap water	29 (7.6)	351(92.4)	
B	Others private tap water^*∗*^			For households with no private tap water
C	Public tap water	0	351(100) ^*∗∗*^	
D	River^*∗*^			
E	Spring^*∗*^			
F	Pond/rain^*∗*^			

Absence or presence was checked (Figures 1–3), but it was not possible to enumerate and calculate the percentages; ^*∗∗*^was implicated as no functional public taps were available in the town during the study.

**Table 6:** Defecation areas, plans, reasons for not having latrine, and problems faced by participants of Negele town (*n* = 208) who had no latrine before and during the study period.

Variable	Frequency (%) of participants per zone			Overall (%)
	Center	East	West	
*Defecation areas*				
Open space	20 (23.8)	26 (30.2)	10 (26.3)	56 (26.9)
Public toilet	0 (0)	0 (0)	0 (0)	0 (0)
In bush	16 (19.0)	29 (33.7)	9 (23.7)	54 (26.0)
In house compound	42 (50.0)	22 (25.6)	16 (42.1)	80 (38.5)
Any place as needed	6 (7.2)	9 (10.5)	3 (7.9)	18 (8.6)

*Reasons for lack of latrine*				
Not knowing the importance of latrine	0 (0)	0 (0)	0 (0)	0 (0)
Lack of enough land	41 (48.8)	35 (40.7)	18 (47.4)	94(45.3)
Lack of enough money	36 (42.9)	37 (43.0)	12 (31.6)	85 (40.8)
Other	7 (8.3)	14 (16.3)	8 (21.0)	29 (13.9)

*Problems faced due to lack of latrine*				
Lack of safety	21 (25.0)	25 (29.1)	14 (36.8)	60 (28.8)
Infectious disease	12 (12.3)	31 (36.0)	9 (23.7)	52 (25.0)
Pollution of living area	32 (38.1)	13 (15.1)	6 (15.8)	51 (24.6)
Moving out in the darkness	19 (22.6)	17 (19.8)	9 (23.7)	45 (21.6)

*A person advised constructing a latrine*				
Health extension	17 (20.2)	27 (31.4)	18 (47.4)	62 (29.8)
Local leader	23 (27.4)	13 (15.1)	5 (13.1)	41 (19.7)
Nobody	44 (52.4)	46 (53.5)	15 (39.5)	105 (50.5)

*Having plan to contact latrine*				
Yes	34 (40.5)	25 (29.1)	9 (23.7)	68 (32.7)
No	50 (59.5)	61 (70.9)	29 (76.3)	140 (67.3)

**Table 7:** Key informants' response regarding latrine and water service coverage.

Variables	Frequency (%) of participants		Overall (%)
	Male	Female	
*Presence of public toilet*			
Yes	0 (0)	0 (0)	0 (0)
No	34 (100)	6 (100)	40 (100)

*Effects of lack of public toilet*			
Suffering to use open defection	15 (44.1)	2 (33.3)	17 (42.5)
Health problems related to waterborne diseases	12 (35.3)	2 (33.3)	14 (35.0)
Environmental pollution	7 (20.6)	2 (33.3)	9 (22.5)

*Factors contributing to lack of latrine*			
Low involvement of administration	11 (32.4)	2 (33.3)	13 (32.5)
Knowledge and attitude-related problems	13 (38.2)	2 (33.3)	15 (37.5)
Shortage of income	5 (14.7)	1 (16.7)	6 (15.0)
Lack of enough land	3 (8.7)	0 (0)	3 (7.5)
Lack of follow-up	2 (6.0)	1 (1.7)	3 (7.5)

*Consequences of lack of latrine*			
Moving out in the dark for defecation	18 (52.9)	3 (50.0)	21(52.5)
Women and girls lack safety and privacy	11 (32.4)	1 (16.7)	12 (30.0)
Suffering from bad odor when defecating around home	5 (14.7)	2 (33.3)	7 (17.5)

*Factors hindering water service coverage*			
Shortage of water from the source and lack of water tanks	9 (26.5)	0 (0)	9 (22.5)
Nonfunctioning of public pipe	14 (41.2)	4 (66.7)	18 (45.0)
Low involvement of administration	8 (23.5)	2 (33.3)	10 (25.0)
Low income of the households	3 (8.8)	0 (0)	3 (7.5)

*The administration planned to improve latrine and tap water coverage*			
Yes	11 (32.4)	2 (33.3)	13 (32.5)
No	23 (67.6)	4 (66.7)	27 (67.5)

*The extent to which the administration planned to raise latrine and tap water coverage*			
26–50	1 (3.0)	0 (0)	1(2.5)
51–80	6 (17.6)	1 (16.7)	7 (17.5)
81 & above	4 (11.8)	1 (16.7)	5 (12.5)
No response	23 (67.6)	4 (66.7)	27 (67.5)

**Table 8:** Water service coverage and related conditions of Negele town in 2018.

Variable	Frequency (%) of participants per zone			Overall (%)
	General	Eastern	Western	
*Having a private tap water*				
Yes	29 (17.5)	0 (0)	0 (0)	29 (7.6)
No	137 (82.5)	126 (100)	88 (100)	351(92.4)

*Source of water if no one has tap water*				
Others' private tap water	39 (28.5)	13 (10.3)	4 (4.5)	56 (16.0)
Nano River	72 (52.5)	83 (65.9)	58 (66.0)	213 (60.6)
Pond and rain	26 (19.0)	30 (23.8)	26 (29.5)	82 (23.4)

*Using protected nontap water*				
Yes	0	0	0	0 (0)
No	137 (100)	126 (100)	88 (100)	351 (100)

*Treating nontap water before drinking*				
Yes, using chemicals like “Bishangari^*a*^ or Wuhagar^*b*^”	15 (11.0)	3 (2.4)	3 (3.4)	21 (6.0)
Yes, boiling	28 (20.4)	26 (20.6)	21 (24.0)	75 (21.4)
No	94 (68.6)	97 (77.0)	64 (72.6)	255 (72.6)

*Knowledge of the effect of impure water on health*				
Yes	82 (60.0)	56 (44.4)	55 (62.5)	193 (55.0)
No	55 (40.0)	70 (55.6)	33 (37.5)	158 (45.0)

*Self or family member exposed to waterborne disease*				
Yes	60 (43.8)	67 (53.2)	53 (60.2)	180 (51.3)
No	77 (56.2)	59 (46.8)	35 (39.8)	171 (48.7)

^*a*^Chlorine-based water treatment solution; ^*b*^a mixture of aluminum sulfate and calcium hypochlorite solution, both are available in local markets and people are advised and encouraged to use them to treat water at home.

**Table 9:** Association of having latrine and various factors (*X*^2^ = Chi-square).

Factor	Description	Have latrine		*X* ^2^	*P* value
		Yes	No		
Zone	Central	82	84	15.180	^*∗*^
	Eastern	40	86		
	Western	50	38		
Sex	Male	112	130	0.279	0.598
	Female	60	78		
Age	18–40	65	123	17.177	^*∗*^
	41–60	79	62		
	61–80	28	23		
Religion	Muslim	142	185	3.197	0.074
	Christian	30	23		
Educational status	No formal education	61	57	10.616	^*∗∗*^
	Primary (1–8)	75	121		
	Secondary (9–12)	21	23		
	>Grade 12	15	7		
Marital status	Single	52	48	8.891	^*∗∗*^
	Married	106	154		
	Widow	14	6		
	Divorced	0	0		
Family size	1–4	85	142	14.23	^*∗*^
	5–10	63	45		
	>10	24	21		
Occupation	Farmer	75	101	4.250	0.373
	Merchant	51	69		
	Government employee	22	19		
	Daily laborer	7	7		
	Other	17	12		

^*∗*^^,^^*∗∗*^Values show significant associations at *p* ≤ 0.01 and *p* ≤ 0.05, respectively.

**Table 10:** Association of having tap water and various factors (*X*^2^ = result of Chi-square).

Factor	Description	Having private tap water	Not having private tap water	*X* ^2^	*P* value
Zone	Central	29	137	40.447	^*∗*^
	Eastern	0	126		
	Western	0	88		
Sex	Male	19	223	0.046	0.831
	Female	10	128		
Age	18–40	8	180	10.452	^*∗*^
	41–60	12	129		
	61–80	9	42		
Religion	Muslim	27	300	1.300	0.254
	Christian	2	51		
Educational status	No formal education	3	115	11.882	^*∗*^
	Primary (1–8)	17	179		
	Secondary (9–12)	8	36		
	>Grade 12	1	21		
Marital status	Single	2	98	8.947	^*∗∗*^
	Married	27	233		
	Widow	0	20		
	Divorced	0	0		
Family size	1–4	9	218	15.095	^*∗*^
	5–10	11	97		
	>10	9	36		
Occupation	Farmer	10	166	7.763	0.101
	Merchant	8	112		
	Governmental	6	35		
	Daily laborer	3	11		
	Other	2	27		

^*∗*^^,^^*∗∗*^Values show significant associations at *p* ≤ 0.01 and *p* ≤ 0.05, respectively.

## Data Availability

All data generated or analyzed during this study are included in this article.

## Supplementary Materials

Supplementary Materials The supplementary material contains a questionnaire, interview, and observation checklists employed for data collection.
