# Supplementary material for: Water and Latrine Services and Associated Factors among Residents of Negele Town, Southeast Ethiopia: A Cross-Sectional Study
Source: J Environ Public Health. 2022 Jan 25;2022:1203514. doi: 10.1155/2022/1203514 (PMC8808149; doi:10.1155/2022/1203514)
Supplement: Supplementary Materials — The supplementary material contains a questionnaire, interview, and observation checklists employed for data collection. [file 1203514.f1.docx]

**Supplementary File**

**A. Questionnaire for households**

My name is Girma Deboch Geleta, M.Sc candidate at MaddaaWalabu University. I am conducting a research entitled “A cross-sectional investigation of Latrine and pure water coverage and related factors among residence of Negele town, Arsi zone, southeast Ethiopia” together with Dr. Diriba Temesgen. The study is my MSc research project approved by Madda Walabu University Biology Department and Office of Post Graduate.

The study is expected to provide information on the Latrine and pure water coverage and related factors of the residents of the town which can be used by stakeholders to plan and take appropriate action(s). So, you are requested to provide genuine response to the given question so that correct information will be gathered. The information that you provide will be used only for the research purpose and kept confidentially. If you do not want to participate in this study for any reason, feel free to not to start or not to continue participating. Do not write your name on the questionnaire paper. Read the questions carefully and encircle the letters for multiple choice questions, and write your answer on the provided answer for open ended questions.

Thank you in advance for willing to participate in this study expending your precious time

April-May, 2018

1. Sex

A. Male

B. Female

2. How old are you?

A. 18-30

B. 31-40

C. 41-80

3 What is your administrative zone in Negele town?

A. Easten

B. Centeral

C. Western

4. In which village are you living in Negele town?

A. Sheep site

B. Najate

C. Center of town

D. Central area

E. Western Hindy

F. Mosque area

5. What is your religion?

A. Christian

B. Muslim

C. other

6. What is your educational status?

A. No formal education (Illiterate) C. Secondary (9-12)

B. Primary (grade 1- 8) D. Greater than grade 12

7. What is your marital status?

A. Single

B. Married

C. Widow

D. Divorced

8. What is your family size?

A. 1-4

B. 5-10

C. greater than 10

9. What is your occupation?

A. Farmer

B. Merchant

C. Governmental employee

D. Daily laborer

E. other

6. Have you a latrine?

A. Yes

B . .No

7. If your answer to Q6 is “NO” where do you and your family defecate?

A. open spaces

B. public toilet

C. in bush

D. in house fence

E. any place as needed

8. If your answer to Q6 is “NO”, why didn’t you have latrine?

A. I do not know the importance of latrine

B. I have no enough land to build it

C. I have no money to build it

D/ other

9. If your answer to Q6 is “NO”, what problems had you faced due to your lack of latrine?

----------------------------------------------------------------------------------------------------------------------------------------------------------------------------------------------------------------------------------------

10. If your answer to Q6 “NO”, has anybody advised you to construct and use latrine? If yes who?

----------------------------------------------------------------------------------------------------------------------------------------------------------------------------------------------------------------------------------------

11. If your answer to Q6 “NO”, do you have a plan to construct and use latrine in the near future?

A. Yes

B. No

12. If you have latrine (if your answer to Q6 is “yes”) what is the number of people using it?

A. 1-5

B. 6-10

C. greater than 10

13. If you have latrine (if your answer to Q6 is “yes”), how far is your latrine from kitchen?

A. 2m-3m

B. 3-5m

C. 6m

D. greater than 6m

14. If you have latrine (if your answer to Q6 is “Yes”), does the latrine have roof?

A. Yes

B. No

15. If you have latrine (if your answer to Q6 is “yes”), does the latrine have door?

A. Yes

B. No

16. If you have latrine (if your answer to Q6 is “yes”), does the latrine have hole cover?

A. Yes

B. No

17. If you have latrine (if your answer to Q6 is “yes”), does the latrine have water supply to clean?

A. Yes

B. No

18. If you have latrine (if your answer to Q6 is “yes”), is there sewerage service in the town to clean it when it is full?

A. Yes

B. No

19. If you have latrine (if your answer to Q6 is “yes”), is there any time in the past when your latrine became full and overflow?

A. Yes

B. No

20. If your answer to Q19 is yes, what measure (s) did you take when your latrine is full and overflown?

--------------------------------------------------------------------------------------------------------------------------------------------------------------------------------------------------------------------------------------------------------------------------------------------------------------------------------------------------------------------------------------------------------------------------------------------------------------------------------

21. If you have latrine, how do you rate it in general?

A. Clean, standardized and good for health

B. Usually dirty and unsuitable for health

22. Do you have pure drinking water source/tap water?

A. Yes

B. No

23. If your answer to 22 is “No", what source of water do you use?

----------------------------------------------------------------------------------------------------------------------------------------------------------------------------------------------------------------------------------------

24.If your answer to Q22 is “No", is your water source protected from possible contamination with human excreta?

A. Yes

B. No

25. Do you treat the non-tap water before drinking?

A/ yes using chemicals like" Bishangari or wuha agar"

B Yes, I boil it

C/No

26. Do you know drinking impure water causes health problem?

A. Yes

B. No

27. Have you or members of your family ever been sick due to drinking impure water?

A. Yes

B. No

**B. Key informant interview**

**1.** Sex --------------

2. How old are you?

--------------------------------------------------------------------------------------------------------------------

3. In which administration zone are you living in Negele town?

--------------------------------------------------------------------------------------------------------------------.

4. In which village of Negele town are you living?

------------------------------------------------------------------------------------------------------------------

5. . What is your religion?

--------------------------------------------------------------------------------------------------------------------

6. What is your marital status?

--------------------------------------------------------------------------------------------------------------------

7. What is your educational status?

--------------------------------------------------------------------------------------------------------------------

8. What is your occupation? ----------------------------------------------------------------------------------

9. Is there public toilet in Negele town? If no what are the influences of absence of public toilet on human and environmental sanitation?

----------------------------------------------------------------------------------------------------------------------------------------------------------------------------------------------------------------------------------------

10. What are the factors that hinder some residents of Negele town not to have their own toilet?

----------------------------------------------------------------------------------------------------------------------------------------------------------------------------------------------------------------------------------------

11. What specific problems have the residents of Negele town encountered due to lack of toilet?

------------------------------------------------------------------------------------------------------------------------------------------------------------------------------------------------------------------------------------------------------------------------------------------------------------------------------------------------------------

12. What are the factors that hinder tap water/pure drinking water coverage the town?

------------------------------------------------------------------------------------------------------------------------------------------------------------------------------------------------------------------------------------------------------------------------------------------------------------------------------------------------------------

9. Does the administration of the town have a plan to improve the latrine and tap water/pure drinking water coverage of the town in the near future? If yes, when and to what percentage?

10. Do you have any relevant additional information to add? (For the chief administration of the town)

Thank you for your cooperation

**C. Observation check list used to collect data**

| No | What were observed  (present/absent if applicable) | Yes (%) | No (%) | Remark |
| --- | --- | --- | --- | --- |
|  |  |  |  |  |
| 1 | Household latrine |  |  |  |
| A | Latrine door |  |  | For households that owned latrine |
| B | Latrine roof |  |  |  |
| C | Latrine hole cover |  |  |  |
| D | Latrine Water supply to clean |  |  |  |
| E | distance of Latrine from kitchen  < 6m |  |  |  |
| 2 | Using public latrine |  |  | for households without latrine |
| 3 | Practicing open defecation |  |  | Various parts of the town were observed |
| 4 | Drinking Water source (n=380) |  |  |  |
| A | Own private tap water |  |  |  |
| B | Others private tap water |  |  | for households with no private tap water |
| C | Public tap water |  |  |  |
| D | River |  |  |  |
| E | Spring |  |  |  |
| F | Pond/rain |  |  |  |
